# Ruptured right Valsalva sinus into the right atrium due to infective endocarditis: a case report

**DOI:** 10.11604/pamj.2020.37.65.21491

**Published:** 2020-09-16

**Authors:** Ayoub Abetti, Thomas Gandet, Abdul Aziz Al Amri, Marouane Ouazzani Ibrahimi, Philippe Rouviere, Alexandra Meilhac, Audrey Agullo, Roland Demaria, Jean Marc Frapier, Bernard Albat

**Affiliations:** 1 Department of Cardiovascular Surgery, Arnaud de Villeneuve Hospital, University Hospital of Montpellier, Montpellier, France,; 2 Department of Cardiovascular Surgery, Mohammed VI University Medical Center, Mohammed the First University Oujda, Oujda, Morocco,; 3 Department of Cardiology, Arnaud de Villeneuve Hospital, University Hospital of Montpellier, Montpellier, France

**Keywords:** Rupture, right Valsalva sinus, Valsalva sinus aneurysm, infective endocarditis

## Abstract

Rupture of Valsalva sinus remains a very rare and deadly complication of Valsalva sinus aneurysm with a high mortality rate. We report here the case of a 47-year-old man who presented to the emergency department with acute exercise-induced dyspnea, chest pain, and fever. Transthoracic (TTE) and transesophageal echocardiography (TEE) highlighted a rupture of the right Valsalva sinus in the right atrium due to infective endocarditis. After stabilization of the patient, a successful surgical repair with double pericardial patches was performed.

## Introduction

Despite advances in surgical techniques, rupture of Valsalva sinus (RVS) is still defined as an exceptional and life-threatening complication of Valsalva sinus aneurysm (VSA) with significant mortality and morbidity [1]. This case reports an acute rupture of the right Valsalva sinus in the right atrium following an infective endocarditis. We also review the literature with a particular focus on its key features, treatment and outcomes.

## Patient and observation

**Figure 1:**
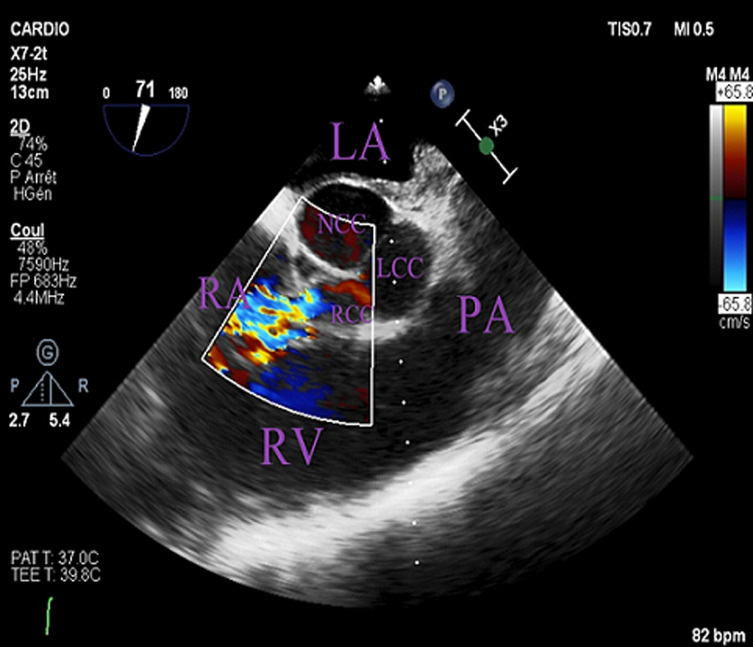
echographic image showing the passage of blood flow from the right Valsalva sinus to the right atrium

**Figure 2:**
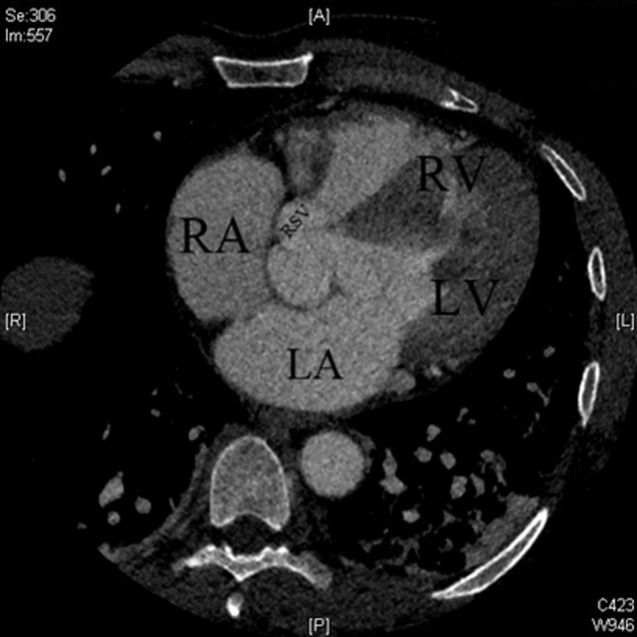
thoracic CT angiography in axial section, showing a passage of contrast material through the right coronary sinus to the right atrioventricular junction

A 47-year-old male, with no pathological history and no significant cardiovascular risk factors presented at the emergency department for the management of chest pain, New York Heart Association Functional (NYHA) stage IV dyspnea and fever at 39°C. At the admission, the patient presented dyspnea associated with chills. His heart sounds were regular with systolic and diastolic murmurs at the aortic area. Pleuro-pulmonary auscultation revealed the presence of bibasilar crackles. Moreover, electrocardiogram test (ECG) found a regular sinus rhythm with a sub-shift of the ST segment in the lateral leads. TTE found a preserved ejection fraction with left ventricular hypertrophy as well as left to right shunt at the level of the aortic valve without apparent origin and normal size and function of right heart cavities. Ascending aorta was moderately dilated (44 mm) with dry pericardial sac. TEE was quickly performed to characterize the observed shunt and showed a rupture of the right Valsalva sinus in the right atrium with a channel of 6 mm (Figure 1). Furthermore, a small 6 mm vibrate image on the right side of the right atrium was also observed indicating the presence of possible infective endocarditis. A computerized tomography (CT) scan was performed and showed the presence of aneurysmal dilatation of the Valsalva sinus (44 mm) with an iodine contrast leakage through the right coronary sinus to the right atrioventricular junction (Figure 2). Blood cultures, serology for Bartonella, Brucellosis, Q fever and syphilis and cytobacteriological examination of the urine were all negative. Dental examination found multiple dental infectious locations that required extractions.

**Figure 3:**
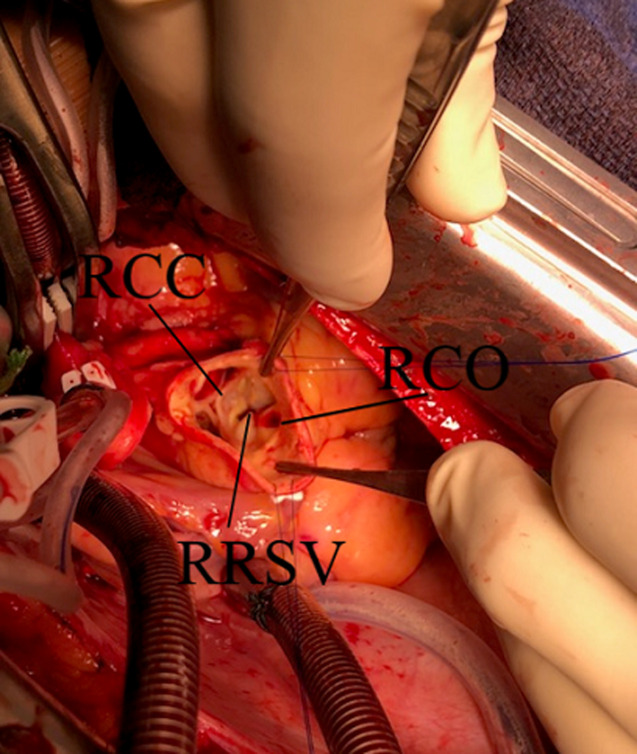
surgical view showing the rupture of the right sinus of Valsalva after complete transverse aortotomy

**Figure 4:**
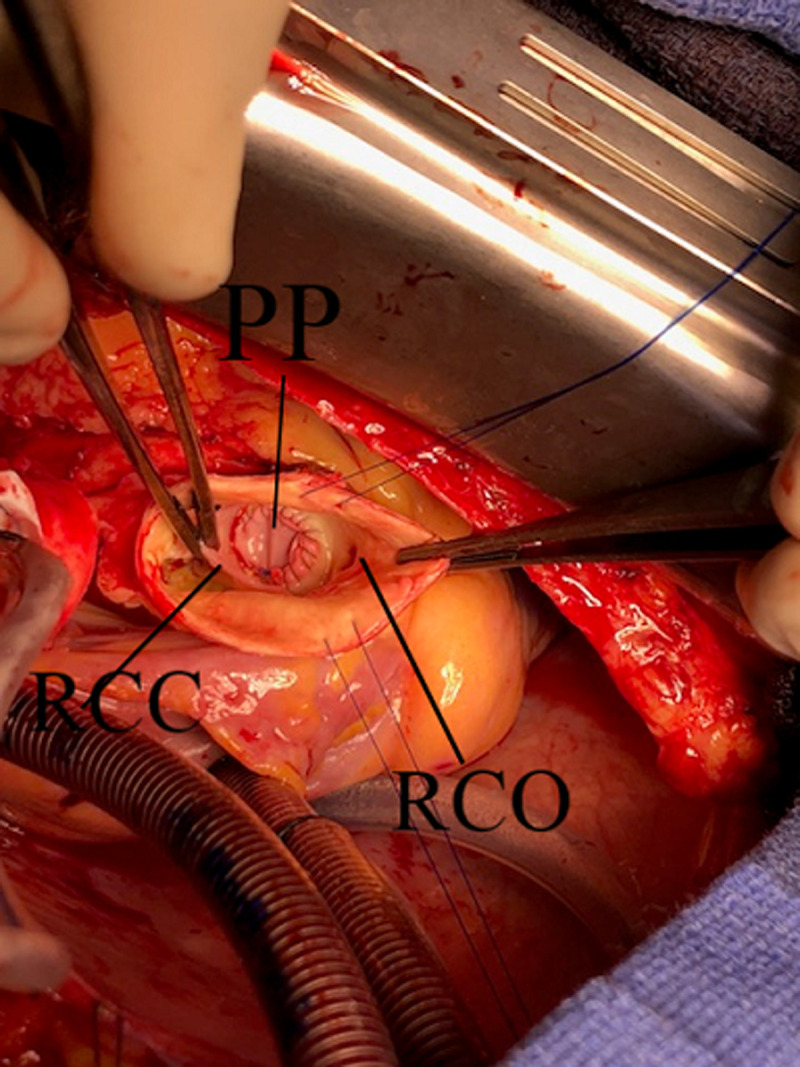
surgical view showing the final result after pericardial patch repair

The optimal care management was discussed with a multidisciplinary team, which recommended intravenous antibiotics including amoxicillin, cefazolin and gentamicin for 14 days before surgical intervention and then an oral switch with amoxicillin andcefazolin for 4 additional weeks. After two weeks of treatment, surgery was performed using a vertical median sternotomy, an extracorporeal circulation and a complete transverse aortotomy until the sino-tubular junction. Peroperative assessment highlighted a tricuspid aortic valve with thin leaflets, dilatation of the right Valsalva sinus with presence of a large perforation (about 1 cm) at the base of the aortic annulus and under the ostium of right coronary artery (Figure 3). Right atriotomy was performed in order to assess the perforation which was localized near to the tricuspid ring at the level of the anteroseptal commissure. After trimming, the orifice was closed using a pericardial patch passing by the aortic way (Figure 4). Vegetations were sent to bacteriological analysis. In order to reinforce the border between the two cavities, another pericardial patch was used using right atrial way. The patient was extubated six hours later and discharged after ten postoperative days. The bacteriological examination of the vegetations based on culture and polymerase chain reaction (PCR) was negative. However, the patient was asymptomatic with no signs of recurrence on echocardiography at the third month of follow up.

## Discussion

RVS is a very rare and serious complication of VSA associated with high morbidity and mortality rates. The survival of untreated patients is about 2 years [1]. VSA is an extremely rare disease with varied incidence from 0,09% to 0,15% across countries [2]. This could be congenital, due to localized dystrophy of the connective tissue in the Valsalva sinus, or an acquired form secondary to atherosclerosis [3], syphilis [3], cystic medionecrosis [3], Behcet's disease [4], Marfan syndrome [5], trauma [3] or infective endocarditis [3]. In our case, RVS was secondary to infective endocarditis. Sinus rupture affects habitually the right coronary sinus (76.8-83.3%), more rarely the non-coronary sinus and unusually the left coronary sinus [6]. Aneurysms of the right coronary sinus may be ruptured in the right ventricle or right atrium which caused left to right shunt [7]. Consequently, this complication is the main prognostic factor. Indeed, acute respiratory failure due to cardiogenic pulmonary edema and acute chest pain due to coronary insufficiency are consequences of a massive rupture of the aneurysm [8]. However, clinical presentation could be more insidious with asymptomatic patients or progressive functional signs, in case of sub acute rupture [9]. According to the current literature, rupture of Valsalva sinus aneurysms could be diagnosed using both TTE and TEE [10]. In our case, TTE showed the presence of a left to right shunt without precise localization, while TEE was able to locate precisely the rupture in the right coronary sinus.

Surgical intervention is recommended when VSA is ruptured or symptomatic. However, surgical approaches must be defined in case of asymptomatic VSA. The main objective of surgical treatment is to remove the aneurysm sac, entirely close the fistula with excluding the two cavities without residual aortic leak or altered conductivity. This intervention involved direct closure of the fistula or closure using a patch placement [11]. In some case series, direct surgical closure was found to be correlated with rupture recurrence and aggravation of aortic leakage [12]. Several authors recommend the use of a patch to close the ruptured VSA in all cases in order to avoid deformation of the aortic valve and to reduce pressure on the sutures [12]. The surgical procedure can be performed by aortic approach right atrial approach, or both [13]. The aortic way allowed better localization of the rupture and its relationship with the coronary ostia and the aortic valve. This approach leads to an easier closure of the fistula without reaching the coronary and the aortic valve. Thus, valve replacement is easy to perform in case of aortic valve damages without the possibility of surgical repair. However, this way does not enable the repair of inter-ventricular communications frequently associated with VSA ruptured [14]. The atrial way should be used only for patients with no significant aortic leakage. Basically, the rupture is closed through the right atrium while leaving the aneurysmal sac in contact with the aorta. This will expose the patient to the risk of infective endocarditis, thrombus development, and recurrent ruptures [15]. In the other hand, combined approaches take more time but allow better closure of the rupture from both sides without leaving any communication between the aneurysmal sac and the blood [3,13]. In our case report, we have closed the ruptured VSA using aortic and right atrial ways based on two patches. This combined procedure allows a complete repair. In addition, using two patches support better the separation of the two cavities and therefore, a significant reduce the risk of recurrence. When ruptured VSA is diagnosed, urgent surgical treatment should be considered, with an usually low operative mortality (0.5 to 11%) and good prognostic outcomes [12,13]. According to Anguera et al. mortality rate may reach to 40% in case of aorto-atrial fistula following infective endocarditis [16]. In this last report [16], risk factors of high mortality are mainly presented by occurrence of heart failure, prosthetic infective endocarditis, and urgent surgery. Our therapeutic attitude was to postpone surgery and stabilize the patient´ hemodynamic and septic clinical conditions before any surgical repair.

## Conclusion

The acute RVS is a very rare and serious complication of Valsalva sinus aneurysm which still has important morbidity and mortality rates. TTE and TEE are the exams of choice for accurately diagnose possible RVS and its associated complications. In the case of RVS due to infective endocarditis, surgical treatment seems to give good results after antibiotics treatment prior to surgery.
